# Pupillary Response to Postural Demand in Parkinson’s Disease

**DOI:** 10.3389/fbioe.2021.617028

**Published:** 2021-04-27

**Authors:** Melike Kahya, Kelly E. Lyons, Rajesh Pahwa, Abiodun E. Akinwuntan, Jianghua He, Hannes Devos

**Affiliations:** ^1^Hinda and Arthur Marcus Institute for Aging Research, Harvard Medical School, Boston, MA, United States; ^2^Department of Neurology, School of Medicine, University of Kansas Medical Center, Kansas City, KS, United States; ^3^Office of the Dean, School of Health Professions, University of Kansas Medical Center, Kansas City, KS, United States; ^4^Department of Physical Therapy and Rehabilitation Science, School of Health Professions, University of Kansas Medical Center, Kansas City, KS, United States; ^5^Department of Biostatistics and Data Science, University of Kansas Medical Center, Kansas City, KS, United States

**Keywords:** pupillary response, posture, balance, vision, dual tasking, Parkinson’s disease

## Abstract

**Background:** Individuals with Parkinson’s disease (PD) may need to spend more mental and physical effort (i.e., cognitive workload) to maintain postural control. Pupillary response reflects cognitive workload during postural control tasks in healthy controls but has not been investigated as a measure of postural demand in PD.

**Objectives:** To compare pupillary response during increased postural demand using vision occlusion and dual tasking between individuals with PD and healthy controls.

**Methods:** Thirty-three individuals with PD and thirty-five healthy controls were recruited. The four conditions lasted 60 s and involved single balance task with eyes open; single balance task with eyes occluded; dual task with eyes open; dual task with eyes occluded. The dual task comprised the Auditory Stroop test. Pupillary response was recorded using an eye tracker. The balance was assessed by using a force plate. Two-way Repeated Measures ANOVA and LSD *post-hoc* tests were employed to compare pupillary response and Center of Pressure (CoP) displacement across the four conditions and between individuals with PD and healthy controls.

**Results:** Pupillary response was higher in individuals with PD compared to healthy controls (*p* = 0.009) and increased with more challenging postural conditions in both groups (*p* < 0.001). The *post-hoc* analysis demonstrated increased pupillary response in the single balance eyes occluded (*p* < 0.001), dual task eyes open (*p* = 0.01), and dual task eyes occluded (*p* < 0.001) conditions compared to single task eyes open condition.

**Conclusion:** Overall, the PD group had increased pupillary response with increased postural demand compared to the healthy controls. In the future, pupillary response can be a potential tool to understand the neurophysiological underpinnings of falls risk in the PD population.

## Introduction

Falls are a common problem for individuals with Parkinson’s disease (PD). A fall is defined as an event in which an individual comes to rest involuntarily on a lower surface, such as the ground or floor (Kellogg, 1987). It has been reported that 50–68% of the PD population fall annually (Huse et al., 2005), which is three times more often than the fall rate of the older population in general (Lord et al., 1993). In addition, 67% of fallers in the PD population have had more than one fall since diagnosis (Contreras and Grandas, 2012). The increased rate of falls suggests that individuals with PD have impaired skills to accurately react and initiate appropriate compensatory postural strategies to prevent falls.

Falls are not only associated with physical function and well-being, but they also share a strong association with cognitive function (Halliday et al., 2018). Higher order executive skills, such as shifting attention, cognitive flexibility, and inhibition, are needed to initiate appropriate postural control strategies (Liu-Ambrose et al., 2008). Studies have shown that fallers with and without PD exhibit increased prefrontal hemodynamic activation while performing walking and cognitive tasks at the same time (Maidan et al., 2016; Verghese et al., 2017). This increased hemodynamic activation is associated with performance on executive tasks (Ranchet et al., 2020). In addition, studies have shown that individuals who have lower cognitive scores on executive function and attention tasks are more likely to fall up to three times than those with higher cognitive scores (Herman et al., 2010). It is possible that cognitive functioning mediates the relationship between reduced postural control and falls in older adults and individuals with PD.

One way to stress the brain to assess its capacity is using a dual task paradigm. Most activities of daily living require performing two tasks simultaneously such as standing while talking or processing information. In such dual tasking conditions, upright stance posture is a basic yet essential motor skill to accomplish various motor and cognitive tasks concurrently (Burki et al., 2017). Although maintaining an upright stance posture seems autonomous and effortless in healthy individuals, it may become challenging and cognitively effortful due to the impaired automatic control process in individuals with PD (Kelly et al., 2012). PD pathology affects subcortical pathways leading to impaired automatic control of movement, which is suggested to be accompanied by a compensatory shift to more voluntary cortical control (Wu et al., 2015). In addition, studies have shown that individuals with PD heavily rely on visual feedback to maintain postural control due to impaired proprioception (Tagliabue et al., 2009; Lahr et al., 2015). While the motor contributions to postural control are well-studied in PD, fewer studies have investigated non-motor contributions such as cognition and vision. It is important to investigate the neurophysiological mechanism of impaired postural control associated with visual occlusion and dual tasking to better understand fall risk and to develop appropriate rehabilitation interventions.

Pupillary response is a non-intrusive, real-time neurophysiological measure of cognitive workload (or mental effort). The reliability and validity of pupillary response to measure cognitive workload were established in individuals without and with PD (Steinhauer and Hakerem, 1992; Pomplun and Sunkara, 2003; Kahya et al., 2020). Increased pupillary response due to cognitive workload stems from increased activation of the locus coeruleus (Beatty, 1982; Sirois and Brisson, 2014). The locus coeruleus plays an essential role in the regulation of physiological arousal and cognition (Sara, 2009). When activated, the locus coeruleus sends inhibitory projections to the parasympathetic Edinger-Westphal nucleus. The Edinger-Westphal nucleus subsequently inhibits the sphincter pupillae muscle, resulting in pupil dilation (Beatty and Lucero-Wagoner, 2000). Increased activity of the locus coeruleus also triggers the sympathetic nervous system, which results in additional pupil dilation due to the activation of the dilator pupillae muscle. Both pupillary response and activation of noradrenergic neurons in the locus coeruleus have been shown to increase in a correlated manner with increased cognitive workload (Varazzani et al., 2015). Although locus coeruleus is one of the first areas undergoing degeneration due to the PD pathophysiology (Micieli et al., 1991; Paredes-Rodriguez et al., 2020), dopamine replacement therapy has been shown to restore pupillary response in individuals with PD (Manohar and Husain, 2015). Also, a previous study showed that pupillary response during “ON” medication reflects cognitive workload in individuals with PD (Kahya et al., 2018a). The pattern of pupillary response in PD to cognitive demand was similar to that of healthy controls, suggesting that early PD pathology does not affect the accuracy of pupillary response in challenging cognitive tasks.

In addition, pupillary response to cognitive workload has been shown to be sensitive to changes in postural demand. Pupillary response increased from a single task to dual task balance conditions in healthy young adults (Kahya et al., 2018b). Also, previous work by our group has shown that pupillary response is a reliable and valid tool of cognitive workload during postural demanding tasks in individuals with PD (Kahya et al., 2020). However, it is not known whether pupillary response is different between individuals with PD and healthy controls during increased postural demand. A better understanding of the cognitive workload measured by pupillary response during postural demand in PD may inform more adequate assessment and treatment strategies to mitigate the effect of increased cognitive workload on balance impairments and falls. Therefore, the purpose of this study was to investigate neurophysiological changes, indexed by pupillary response, during postural demanding tasks between individuals with PD and healthy controls. Previous research in the PD population showed that individuals with PD had higher brain hemodynamic activation and increased brain power with increased postural demand compared to healthy controls (Maidan et al., 2016, 2019). Therefore, we hypothesized that individuals with PD would demonstrate higher pupillary response compared to healthy controls. An exploratory aim was to investigate the differences in pupillary response during postural demand between three groups: PD fallers, PD non-fallers, and healthy controls.

## Materials and Methods

Thirty-three individuals with PD and thirty-five age- and sex-matched healthy controls were recruited. Power analysis was performed for sample size estimation based on data from our previous study (Kahya et al., 2018a). The effect size (f) in this study was 0.26, which is considered a moderate effect size based on Cohen’s criteria (Cohen, 1988). Using this effect size, 56 participants (*n* = 28 PD and *n* = 28 healthy controls) were needed to detect a moderate effect size of f = 0.26 with 80% power using a Two-way Repeated-Measures ANOVA, with two groups (between-factor) and four conditions of measurement (within-factor). To account for the possibility of random missing data, we recruited 20% more participants than our sample size calculation. Hence, we recruited 68 (*n* = 33 PD and *n* = 35 healthy controls) participants.

Participants with PD were categorized into fallers (*n* = 14, number of falls > 0) or non-fallers (*n* = 19, number of falls = 0) based on their self-reported fall history in the past 12 months (Lindholm et al., 2016). Patients with PD were recruited from the University of Kansas Medical Center Parkinson’s Disease and Movement Disorder Center between 08/2018 and 02/2019. Diagnosis of idiopathic PD was established according to the United Kingdom Parkinson’s Disease Society Brain Bank Clinical Diagnostic Criteria (Hughes et al., 1992). Healthy controls were the spouse/significant others of the participants with PD or members of the community.

Inclusion criteria for the PD group were (1) voluntary consent, (2) ability to speak and understand the English language, and (3) mild to moderate disease severity (Hoehn and Yahr stage II and III). Exclusion criteria were (1) diagnosis of mild cognitive impairment or dementia, (2) atypical parkinsonism, (3) history of neurological or vestibular conditions unrelated to PD, (4) current visual acuity problems that cannot be resolved by corrective lenses or visual field problems, (5) severe trunk and head dyskinesia or dystonia in the medication “on” state, (6) blepharospasm, (7) deep brain stimulation, (8) unpredictable motor fluctuations, and (9) any musculoskeletal condition that might affect standing and balance activities. Inclusion criteria for the healthy controls were (1) voluntary consent and (2) ability to speak and understand the English language. We excluded individuals who (1) had or currently have neurological or vestibular problems, (2) any musculoskeletal problems that might affect balance activities, and (3) visual acuity problems that cannot be resolved by corrective lenses or visual field problems.

This study was approved by the Human Subjects Committee at the University of Kansas Medical Center. Participants were asked to make one visit to the University of Kansas Medical Center Parkinson’s Disease and Movement Disorder Center. Prior to enrollment written informed consent was obtained from all study participants. Study testing lasted for a total of 2 h including consent and breaks. All assessments were done in the medication “on” state. Participants with PD were tested approximately 30–45 min after medication intake to minimize the possibility of wearing-off, which could potentially affect the test results. It is reported that individuals with PD had better recognition of wearing-off based on their self-reported exacerbated motor and non-motor symptoms compared to a PD specialist (Stacy et al., 2005). Therefore, if the medication wore off based on participants’ self-report during the assessment, the assessment was stopped until approximately 30 min after the next medication dose when the participant was again in the medication “on” state. The “on” medication for clinical assessments was defined as the patients taking their normal daily medications in the optimally medicated state, as determined by both the patient and the researcher.

Demographic characteristics and medical history were collected from the participants. A list of prescribed and unprescribed medications was obtained from the participants’ medical records. Levodopa Equivalent Daily dose was calculated to tally antiparkinsonian related medication usage (Deuschl et al., 2006). Global cognitive functioning was measured through the Montreal Cognitive Assessment (MoCA) (Nasreddine et al., 2005). Restrictions in activities of daily living and motor impairments were evaluated through the Movement Disorders Society-Unified Parkinson’s Disease Rating Scale (MDS-UPDRS) Part II (motor experiences of daily living) and Part III (motor examination) (Goetz et al., 2008). The Hoehn and Yahr (H&Y) Scale (Hoehn and Yahr, 1967) was used to assess PD severity. The Scales for Outcomes in Parkinson’s Disease-Autonomic Dysfunction (SCOPA-AUT) (Visser et al., 2004) was conducted to assess autonomic symptoms as dysautonomia may potentially influence pupillary response in PD. Lastly, fear of falling was measured through the Falls Efficacy Scale-International (FES-I).

All participants were asked to wear Tobii Pro 2 glasses (Tobii Technologies, Inc.) to measure pupillary response during the testing. Participants were tested in a room with no windows. The temperature and lighting conditions of the room were identical for each participant. A force plate was used (AMTI OPT464508-1000, Advanced Mechanical Technology, Inc.) to assess Center of Pressure (CoP) displacement with a sampling frequency of 100 Hz. Participants were instructed to stand with their shoes on by placing their feet oriented at 14° with heel centers 17 cm apart. The assessment and testing took around 2 h and we mitigated the effect of fatigue by giving breaks and by allowing participants to have rest periods anytime during the study. Participants were asked to complete the following conditions in randomized order.

1. Single balance eyes open condition: Participants stood on a force plate and were instructed to maintain an upright standing posture for 60 s.
2. Single balance eyes occluded condition: Participants were instructed to stand on a force plate for 60 s while their eyes were occluded with a sleep mask. The sleep mask was placed in front of the eye-tracking glasses. Participants were instructed to keep their eyes open throughout the condition.
3. Dual task eyes open condition: Participants were instructed to stand on the force plate for 60 s while concurrently completing an Auditory Stroop test.
4. Dual task eyes occluded condition: Participants were instructed to stand on a force plate for 60 s while simultaneously completing an Auditory Stroop test with their eyes occluded.

The Auditory Stroop test was shown to be one of the key determinants of dual task performance in individuals with PD (Strouwen et al., 2016). Therefore, the Auditory Stroop test was conducted to stress the executive function and cognitive flexibility abilities of the participants. During the Auditory Stroop test, participants heard the word “high” or “low” in a high or low pitch and were instructed to name the pitch of the stimulus, while ignoring the meaning of the word. Participants heard congruent stimuli where the word and pitch are equal (e.g., “high” at a high pitch) or incongruent stimuli where the word and pitch differ (e.g., “high” at a low pitch) in a random order for 60 s. There were 30 stimuli presented at 2-s intervals for 60 s. Participants were instructed to respond as accurately and as fast as possible. To standardize the test, participants wore headphones and the stimuli were played by a digital recorder.

After testing, the pupillary response data were extracted at 60 Hz from EyeWorks Analyze software. By solely measuring the change of the raw pupil dilation, there are potential limitations such as the light reflex and movement artifacts interfering with the pupil size. We minimized these potential confounders by keeping lighting in the room constant and having participants focus on a picture of dots on the wall to minimize eye movements to better capture pupil dilation. In addition, we used the Index of Cognitive Activity (ICA) algorithm, calculated through the EyeWorks Analyze software to differentiate pupillary response due to workload from the light reflex (Marshall, 2007). In this study, pupil dilation was measured by an eye-tracker, and ICA analysis was conducted to compare cognitive workload across the conditions. This algorithm computes the number of unusual increments in pupil size per second. These values are then transformed into a continuous scale ranging between 0 (no cognitive workload) and 1 (maximum cognitive workload). Based on this algorithm the noisy signals are reduced to nearly zero (Marshall, 2007). The mean ICA was calculated after each condition for all groups.

In addition, the CoP displacement in the anterior-posterior (AP) and medio-lateral (ML) directions were calculated by using NetForce Ver. 3.5.3 software for each condition. We included several functional mobility tests to better understand an individual’s risk of falling and provide a standardized assessment of disability and functional limitations. The APDM Movement Monitoring inertial sensor system (APDM Inc., Portland, OR, United States) was used to objectively characterize balance and gait impairments. After calibration, six synchronized Opal inertial sensors were fitted on each participant via elastic straps [sternum, waist (at the level of the fifth lumbar spine), dorsal surface of bilateral wrists and top of each foot]. Participants were asked to complete the Timed Up and Go (TUG) test and TUG-cognitive (TUG-COG) while wearing the sensors. TUG is a widely used, reliable, and valid test to examine functional mobility and falls risk in individuals with PD (Morris et al., 2001). This test also assesses multiple postural components such as balance control, physical mobility, and gait; therefore, we decided to use this test to better characterize fall risk and to confirm the classification of self-reported fallers and non-fallers. Participants were asked to sit on a chair to start the TUG test and instructed to stand up from the chair, walk 3 m at normal speed, turn back, walk back to the chair, and then sit down. The test was done three times and the average turning and completion time was calculated. It has been shown that both TUG turning duration and TUG completion time provide a good understanding of functional impairments and fall risk in individuals with PD (Mancini et al., 2015). During TUG-COG, individuals were asked to count backward by 7 starting from a random three-digit number while standing up from the chair, walking 3 m at normal speed, turning back, walking back to the chair and then sitting down. The TUG-COG was done three times and average turning and completion times were calculated. Signals were automatically processed and calculated via the corresponding Mobility Lab^TM^ software package.

## Statistical Analysis

Homogeneity of variance between groups was verified using Levene’s test. Independent *t*-tests were used to compare demographic and clinical variables between individuals with PD and healthy controls. One-way Analysis of Variance (ANOVA) was used to compare demographic and clinical variables between PD fallers, PD non-fallers, and healthy controls. Fisher’s exact test was used to compare nominal variables. Independent *t*-tests were used to compare disease-specific variables between PD fallers and PD non-fallers. Two-way Repeated Measures ANOVA and LSD *post-hoc* tests were employed to compare pupillary response and CoP displacement across the four conditions and between individuals with PD and healthy controls. The same test was run to compare pupillary response and CoP displacement between PD fallers, PD non-fallers, and healthy controls. Pearson’s correlation was used to analyze the relationship between pupillary response and CoP displacement. The results were interpreted as follows: >0.70 is strong, 0.50–0.70 is moderate, 0.30–0.50 is weak (Hinkle et al., 1988). All statistical analyses were performed with the IBM SPSS Statistics v.26 software (IBM, Armonk, NY, United States). Bonferroni correction was applied to adjust multiple pairwise comparisons and *p* < 0.01 were considered statistically significant.

## Results

A summary of the demographic and clinical characteristics of two groups are shown in Table 1. Individuals with PD had mild to moderate disease severity (*n* = 24 in H&Y stage II; *n* = 9 H&Y stage III) and MDS-UPDRS II and III scores (Supplementary Table S1). There were no significant differences in demographic variables between the groups except that healthy controls had more years of education. In addition, PD fallers had significantly higher FES-I scores, TUG turning and total time, and TUG-COG turning time compared to PD non-fallers and healthy controls. However, there was no significant difference in the TUG-COG total time between the groups.

**TABLE 1:** Demographic and clinical characteristics.

**Variables**	**PD fallers (*n* = 14)**	**PD non-fallers (*n* = 19)**	**Healthy controls (*n* = 35)**	***p*-value**
Age (years)	69.9 ±6.8	68.8 ± 6.9	68.5 ± 6.2	0.8
Sex (female/male, n)	7/7	7/12	21/14	0.3
Education (years)	15.2 ± 2.2	15.5 ± 2.1	17.3± 3.5	0.02
MoCA [0–30]	26.8 ± 3.8	26.3 ± 2.3	26.6± 2.3	0.8
MDS-UPDRS II [0–52]	14.3 ±8.3	10.1 ±7.9	N/A	0.1
MDS-UPDRS III [0–72]	47.4 ± 12.4	41.5 ± 16.4	N/A	0.3
Modified H&Y scale [1–5]	2.4 ± 0.6	2.2 ± 0.4	N/A	0.2
LED (mg)	312.2± 302.6	294.5± 236.8	N/A	0.9
SCOPA-AUT [0–69]	16.6 ±10.2	14.3 ± 8.2	N/A	0.5
FES-I [16–64]	30.6±11.6	23.3 ±7.5	18.3 ± 2.1	<0.001
TUG turning time (sec)	2.7 ± 0.5	2.6 ± 0.6	2.3 ± 0.3	0.01
TUG total time (sec)	15.1 ± 5.2	13.2 ± 3.2	11.6± 1.8	0.01
TUG-COG turning time (sec)	2.8 ± 0.5	2.6 ± 0.6	2.3± 0.4	0.02
TUG-COG total time (sec)	15.8 ±0.3	17.3 ± 12.1	14.4 ±5.4	0.44

*PD, Parkinson’s disease; MoCA, Montreal Cognitive Assessment; MDS-UPDRS II, Movement Disorder Society Unified Parkinson Disease Rating Scale motor experiences of daily living; MDS-UPDRS III, Movement Disorder Society Unified Parkinson Disease Rating Scale motor examination; H&Y, Hoehn and Yahr; LED, Levodopa Equivalent Dose; SCOPA-AUT, Scales for Outcomes in Parkinson’s Disease-Autonomic questionnaire; N/A, Not Applicable. FES-I, Falls Efficacy Scale-International, TUG, Timed Up and Go; TUG-COG, Timed Up and Go-Cognitive. The results are presented as mean ± standard deviation except for the sex variable. The ranges of each scale were presented in the brackets.*

We first conducted a two-way repeated measures ANOVA with main effects of group (PD vs. controls), condition, and interaction effect of group × condition. Individuals with PD had a higher pupillary response compared to healthy controls (*p* = 0.009). In addition, a significant within-condition effect was observed, indicating that pupillary response increased with increased postural demand (*p* < 0.001). The *post-hoc* analysis demonstrated that pupillary response was significantly larger in the single balance eyes occluded (*p* < 0.001), dual task eyes open (*p* = 0.01), and dual task eyes occluded (*p* < 0.001) conditions compared to single task eyes open condition. No other *post-hoc* within group differences were observed. Lastly, there was a trend in the interaction effect of group × condition (*p* = 0.06), suggesting cognitive workload as a result of postural demand manifests differently in participants with PD compared to healthy controls (Supplementary Figure S1).

**FIGURE 1:**
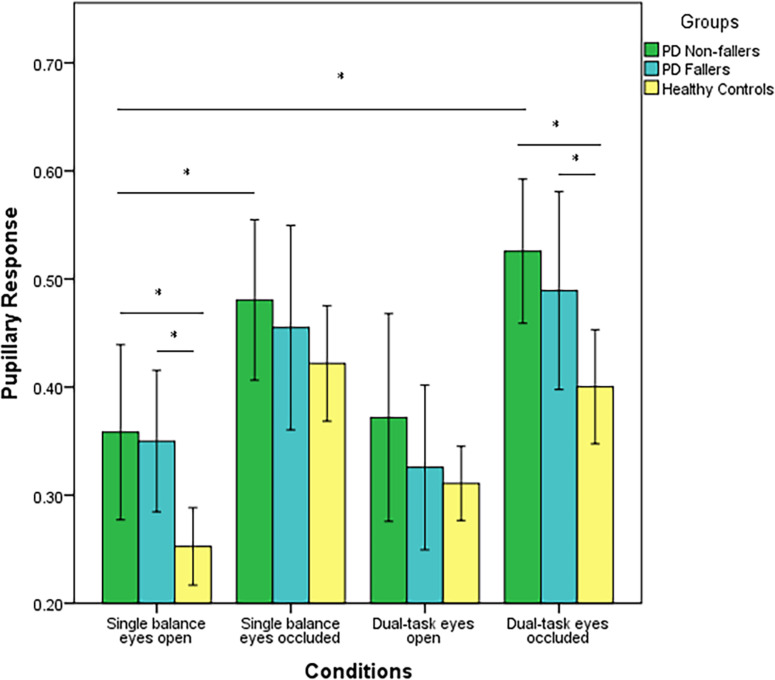
Mean values (range 0–1) and standard error of the mean (SEM) of pupillary response of PD fallers, PD non-fallers, and healthy controls across the conditions. *p < 0.01.

Next, two-way repeated measures ANOVA was employed to compare pupillary response between PD fallers, PD non-fallers, and controls across the four conditions. Pupillary response was significantly different between the groups (*p* < 0.001). The *post-hoc* analysis demonstrated that PD non-fallers (*p* = 0.001) and PD fallers (*p* = 0.01) exhibited greater pupillary response compared to healthy controls over all the conditions. Although there was no significant difference between PD non-fallers and PD fallers between-group grand averages of the four conditions, the comparison of mean and standard deviation demonstrated that PD non-fallers (mean ± s.d.) (0.43 ± 0.2) exhibited greater pupillary response compared to the PD fallers (0.38 ± 0.2) and healthy controls (0.34 ± 0.1) (*p* = 0.25). Pupillary response significantly increased with increased postural demand, especially from eyes open to eyes occluded conditions (*p* < 0.001). However, no interaction effect was observed (*p* = 0.77) (Figure 1).

**FIGURE 2:**
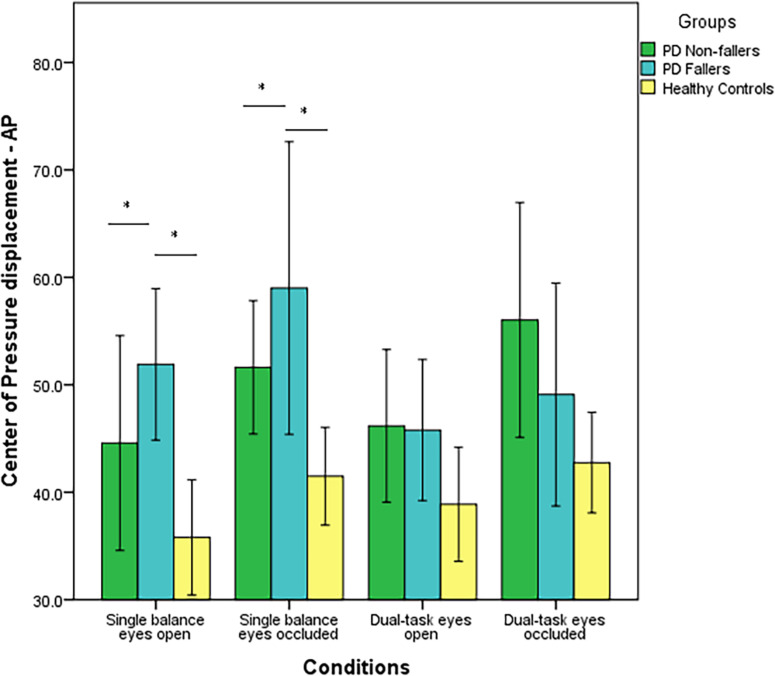
Mean values (in mm^2^/s) and standard error of the mean (SEM) of Center of Pressure (CoP) displacement in the Anterior-Posterior (AP) direction of PD fallers, PD non-fallers, and healthy controls across the conditions. *p < 0.01.

CoP displacement in the AP direction was significantly different between the three groups (*p* < 0.001). The *post-hoc* analysis demonstrated there was a significant difference between PD non-fallers and healthy controls (*p* = 0.001) as well as between PD fallers and healthy controls (*p* = 0.001). However, there was no difference between PD non-fallers and PD fallers (*p* = 0.61). Also, there was not a significant within-condition effect (*p* = 0.04). There was not an interaction effect of group × condition (*p* = 0.48) (Figure 2). Lastly, there were no significant between-group (*p* = 0.25) or within-group (*p* = 0.02) differences for the CoP displacement in the ML direction.

**FIGURE 3:**
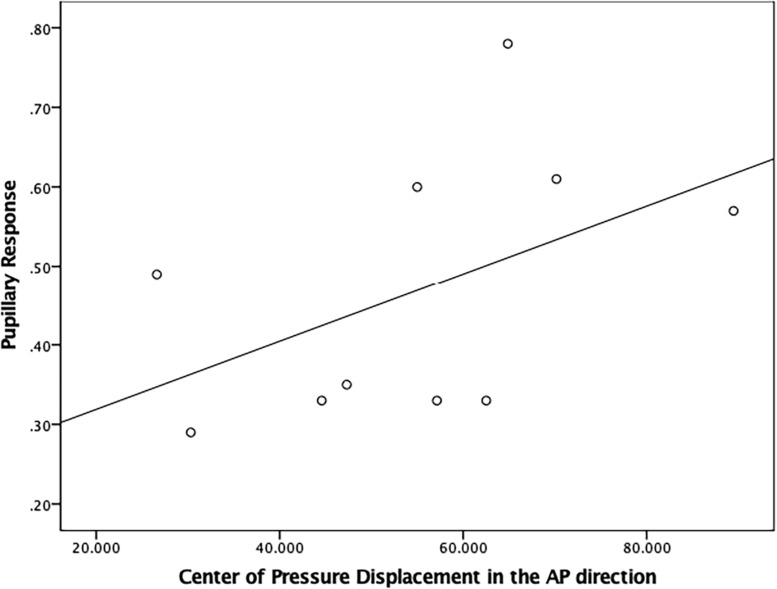
Correlation analysis between pupillary response and Center of Pressure (CoP) displacement (in mm^2^/s) in the Anterior-Posterior direction in PD fallers.

**FIGURE 4:**
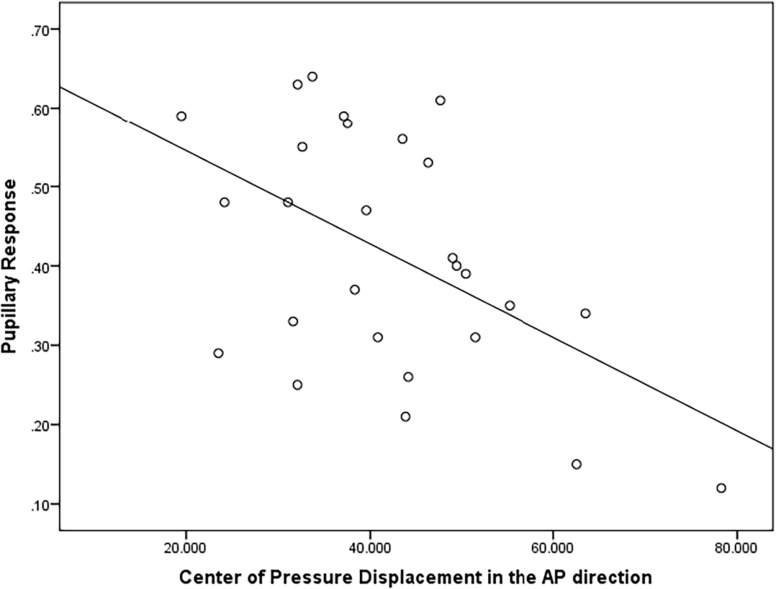
Correlation analysis between pupillary response and Center of Pressure (CoP) displacement (in mm^2^/s) in the Anterior-Posterior direction in healthy controls.

There was a moderately strong, positive, but non-significant correlation (*r* = 0.50; *p* = 0.15) between pupillary response and CoP displacement in PD fallers group during single balance eyes occluded (Figure 3). Also, a moderate negative correlation was observed between pupillary response and CoP displacement in healthy controls during single balance eyes occluded (*r* = −0.51; *p* = 0.006) (Figure 4). No other moderate or strong correlations were observed between pupillary response and COP displacement. Lastly, the Auditory Stroop results demonstrated that both individuals with PD and healthy controls responded correctly to 75% of the questions during the test both in dual task eyes open and dual-task eyes occluded conditions.

## Discussion

To our knowledge, this is the first study that investigated pupillary response as a measure of cognitive workload to changes in postural demand in individuals with PD. The findings of this study demonstrated that, overall, individuals with PD exhibited higher cognitive workload measured by pupillary response compared to healthy controls. In addition, a significant condition effect was observed suggesting that individuals with PD and healthy controls displayed increased pupillary response from single balance eyes open to dual task eyes open condition and to dual task eyes occluded conditions. Our results demonstrate that pupillary response is a sensitive neurophysiological measure of postural demand in both individuals with PD and healthy controls. The results imply that vision occlusion and secondary cognitive task impose additional cognitive workload that can be adequately captured through the pupillary response.

Pupillary response was sensitive to incremental difficulty levels of postural demand in both groups. In addition, the PD group exhibited greater postural demand for all tasks compared to the healthy controls. These findings were similar to previous studies, which measured brain activation by using functional near-infrared spectroscopy (fNIRS) or electroencephalogram during dual-tasking in PD. Studies have shown that individuals with PD had higher brain activation perhaps to compensate for the neurodegeneration compared to healthy older adults (Maidan et al., 2016, 2019). Increased pupillary response in individuals with PD might be related to a neurodegeneration process leading to limited cognitive resources to exert during balance tasks compared to the healthy controls. A greater understanding of the amount of cortical workload involved in balance tasks with PD-related neurodegeneration will allow the development of PD-specific interventions targeting cortical activity and eventually decrease fall risk in these individuals. The novelty of our study is to use pupillary response, which may offer an inexpensive, less intrusive alternative to other neurophysiological tools, such as fNIRS or electroencephalogram, in unraveling brain activation during postural demand in individuals with PD. In the future, pupillary response can be a potential tool to understand the neurophysiological underpinnings of falls risk in the PD population.

Although the results were not significant, PD non-fallers exhibited higher mean pupillary response compared to PD fallers and healthy controls. These results are important to discuss since there is a need to objectively characterize falls risk in clinical practice for individuals with PD. Our results were contradictory to previous studies, which have shown that PD fallers and older adults who are fallers had higher brain activation, measured by fNIRS, in the prefrontal cortex compared to their non-fallers group during dual task gait activities (Halliday et al., 2018; Maidan et al., 2018). One possible explanation is that PD fallers may need to use additional brain networks from the prefrontal cortex as a compensatory strategy to maintain their balance. In our study, we used pupillary response to understand cognitive workload, which has a greater temporal resolution compared to fNIRS (Numata et al., 2019). Therefore, it is possible that pupillary response better corresponds to the timing of the actual brain activity compared to the fNIRS.

PD fallers and PD non-fallers had higher CoP displacement in the AP direction, higher fear of falling, and longer time to complete TUG compared to healthy controls. In the literature, similar results were published. Studies showed higher fear of falling and increased time to complete TUG and TUG-COG in PD fallers compared to PD non-fallers (Vance et al., 2015; Kader et al., 2016). Higher fear of falling is a predictor of future falls and associated with worse motor symptoms and lower quality of life in the PD population (Jonasson et al., 2018). Therefore, it was not surprising that our data demonstrated higher fear of falling and worse outcomes in clinical fall risk assessments in PD fallers. In addition, Matinolli et al. (2007) demonstrated that individuals with PD who are fallers had higher postural sway and CoP displacement compared to PD non-fallers and healthy controls. Other studies demonstrated increased CoP displacement with visual deprivation and additional cognitive load in individuals with PD (Marchese et al., 2003; Holmes et al., 2010; Morenilla et al., 2020). In the present study, Figure 2 demonstrated increased CoP displacement in PD fallers with visual occlusion; however, the effect of additional cognitive load was absent measured by CoP displacement. Also, PD fallers had increased CoP displacement during single tasks but showed decreased displacement during the dual task conditions, whereas PD non-fallers had a similar pattern of CoP displacement compared to healthy controls. This might suggest that PD fallers demonstrated a rigid posture to maintain their balance during dual task activities. In PD, it is typical to observe increased CoP displacement and postural sway during balance but also a high and unadaptable axial tone (rigidity), which both negatively impact postural balance (Cohen et al., 2015). Based on our results, increased rigidity perhaps contributes more to falls, which suggests that PD fallers are unable to react and initiate appropriate compensatory postural strategies to prevent falls. Alternatively, decreased CoP displacement during the dual task conditions can be explained as individuals prioritize and divert their attention to the motor task since during the Auditory Stroop test individuals responded wrongly to 25% of the questions. These results might suggest that both individuals with PD and healthy older adults exhibited cognitive-motor interference resulting in decreased performance in one or both tasks under dual task conditions.

Lastly, it is important to couple behavioral and neurophysiological results to increase our understanding of brain-behavior interaction. A moderate positive correlation was observed between pupillary response and CoP displacement in PD fallers, whereas a moderate negative correlation was observed between pupillary response and CoP displacement in healthy controls during single balance eyes occluded. It is possible that impaired posture control is associated with higher cognitive workload in individuals with PD who are fallers, whereas healthy controls exhibit higher cognitive workload as a compensatory strategy to maintain their posture. Future studies are needed to better understand the relationship between neurophysiological and behavioral results in healthy and disease population.

This study has several limitations. PD fallers and non-fallers were grouped based on their self-report of falls. However, the clinical fall risk assessments demonstrated that PD fallers had significantly higher TUG and TUG-COG completion time and fear of falling compared to PD non-fallers and healthy controls. Therefore, we assume that individuals were assigned to correct groups based on their self-reported falls. In addition, we did not control the number of falls in our analysis in healthy controls. Only five individuals reported a history of falls out of 35 participants; therefore, we assume that history of falls in healthy controls was not a major confounding factor in our results. Although we measured subjects’ cognition by MoCA and years of education as proxies of cognitive capacity, future studies should consider formally measuring cognitive capacity, for example, through the cognitive reserve index questionnaire (Nucci et al., 2012), to better understand the neurophysiological response of the brain to increased postural demand in aging and age-related neurodegenerative conditions. Lastly, during the dual task conditions individuals engaged with triple tasks including balance, cognition, and speaking to respond Auditory Stroop test. It is possible that individuals allocated a small amount of cognitive resources for speaking. However, the main idea of using dual task paradigms is to challenge individuals’ ability to more than one task at the same time. The Auditory Stroop task in combination with a postural test requires a significant cognitive capacity for older adults and individuals with PD. Also, during the Auditory Stroop test individuals only responded by saying “low” or “high.” Therefore, in this study, we believe that individuals did not allocate significant cognitive capacity to speaking.

## Conclusion

Pupillary response is a non-intrusive, objective, and sensitive neurophysiological measure of cognitive workload during postural demand in older adults with and without PD. Individuals with PD exerted greater pupillary response to remain standing still under visual occlusion and dual tasking conditions. In the future, pupillary response can be a potential tool to understand the neurophysiological underpinnings of falls and falls risk in the PD population.

## Data Availability Statement

The raw data supporting the conclusions of this article will be made available by the authors, without undue reservation.

## Ethics Statement

This study was approved by the Human Subjects Committee at the University of Kansas Medical Center. The patients/participants provided their written informed consent to participate in this study.

## Author Contributions

MK: conception, organization, and execution for the research project, design and execution for the statistical analysis, and writing of the first draft for manuscript preparation. KL: conception and organization for the research project, review and critique for the statistical analysis, and manuscript preparation. RP: organization for the research project, review and critique for the statistical analysis, and manuscript preparation. AA: conception for the research project, review and critique for the statistical analysis, and manuscript preparation. JH: conception for the research project, design, review, and critique for the statistical analysis, and review and critique for the manuscript preparation. HD: conception, organization, and execution for the research project, design, review, and critique for the statistical analysis, and review and critique for the manuscript preparation. All authors contributed to the article and approved the submitted version.

## Conflict of Interest

MK holds a fellowship through NIH T32 Harvard Translational Research in Aging Training Program. KL reports consultancies with Abbott and Acorda. RP reports consultancies with Abbott, AbbVie, ACADIA, Acorda, Adamas, Amneal, CalaHealth, Global Kinetics, Impel Neuropharma, Kyowa, Lundbeck, Mitsubishi, Neurocrine, Orbis Bioscience, PhotoPharmics, Prilenia, Sunovion, Teva Neuroscience, US World Meds and research support from Abbott, AbbVie, Addex, Biogen, Biohaven, Boston Scientific, EIP, Global Kinetics, Impax, Lilly, Neuroderm, Neuraly, Parkinson’s Foundation, Pharma 2B, Prelinia, Roche, SIS, Sun Pharma, Sunovion, Theranexus, Theravance, US WorldMeds, and Voyager/Neurocrine. AA has received honoraria for being a member of the National Advisory Board for the NIH National Center for Medical Rehabilitation Research and has royalties from a scientific invention with license held by the University of Kansas Medical Center. HD holds research grants from National Institute for Aging, Parkinson’s Foundation, National Multiple Sclerosis Society, Georgia CTSA, and royalties for the sale of the Portable Driving Simulator. The remaining author declares that the research was conducted in the absence of any commercial or financial relationships that could be construed as a potential conflict of interest.

## Supplementary Material

Supplementary Figure 1 Mean values (range 0–1) and standard error of the mean (SEM) of pupillary response of individuals with PD and healthy controls accross the conditions. **p* < 0.01.

Supplementary Table 1 Demographic and ClinicalCharacteristics. PD = Parkinson’s disease; MoCA = Montreal Cognitive Assessment; MDS-UPDRS II = Movement Disorder Society Unified Parkinson Disease Rating Scalemotor experiences of daily living; MDS-UPDRS III = Movement Disorder Society Unified Parkinson Disease Rating Scalemotor examination; H&Y = Hoehn and Yahr; LED = Levodopa Equivalent Dose; SCOPA-AUT = Scales for Outcomes in Parkinson’s Disease-Autonomic questionnaire; N/A = Not Applicable. FES-I = Falls Efficacy Scale-International, TUG = Timed Up and Go; TUG-COG = Timed Up and Go-Cognitive. The results are presented as mean ± standard deviation except for the sex variable.
