# Supplementary figures and images for: Pupillary Response to Postural Demand in Parkinson’s Disease
Source: Front Bioeng Biotechnol. 2021 Apr 27;9:617028. doi: 10.3389/fbioe.2021.617028 (PMC8111006; doi:10.3389/fbioe.2021.617028)

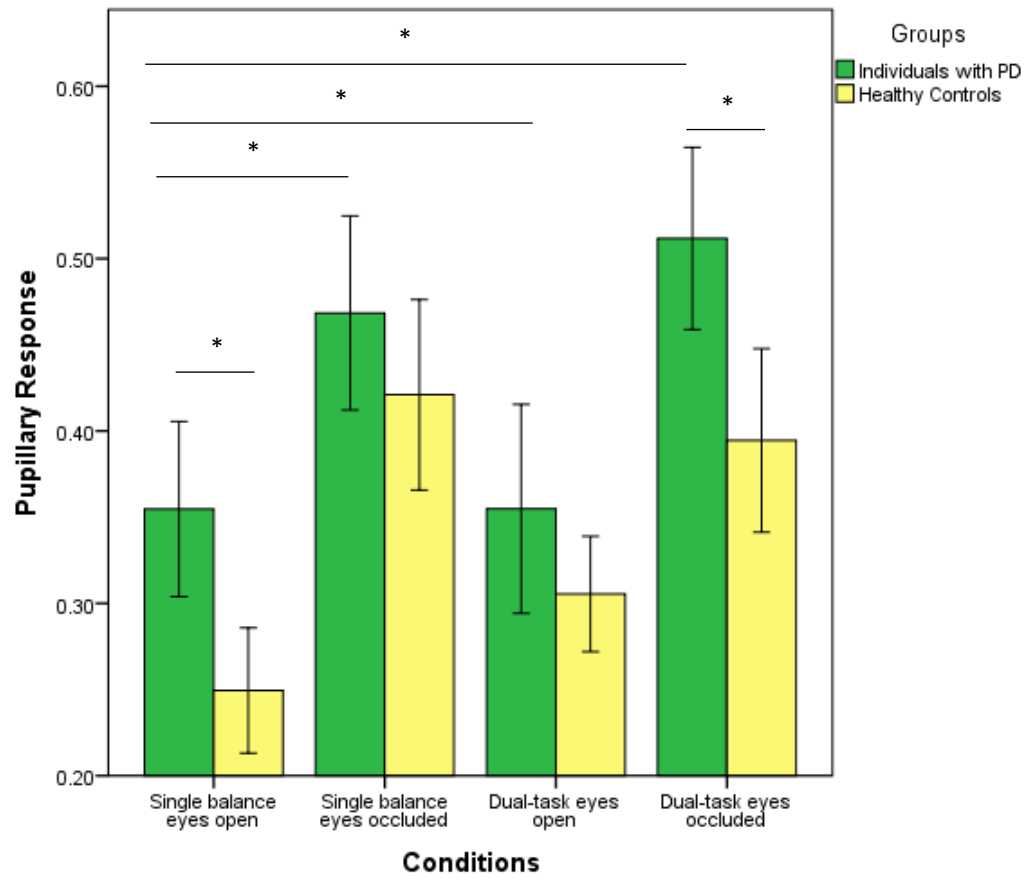

Supplement: Supplementary Figure 1 — Mean values (range 0–1) and standard error of the mean (SEM) of pupillary response of individuals with PD and healthy controls accross the conditions. *p < 0.01. [file Image_1.pdf]
