# Supplementary material for: Pupillary Response to Postural Demand in Parkinson’s Disease
Source: Front Bioeng Biotechnol. 2021 Apr 27;9:617028. doi: 10.3389/fbioe.2021.617028 (PMC8111006; doi:10.3389/fbioe.2021.617028)
Supplement: Supplementary Table 1 — Demographic and ClinicalCharacteristics. PD = Parkinson’s disease; MoCA = Montreal Cognitive Assessment; MDS-UPDRS II = Movement Disorder Society Unified Parkinson Disease Rating Scalemotor experiences of daily living; MDS-UPDRS III = Movement Disorder Society Unified Parkinson Disease Rating Scalemotor examination; H&Y = Hoehn and Yahr; LED = Levodopa Equivalent Dose; SCOPA-AUT = Scales for Outcomes in Parkinson’s Disease-Autonomic questionnaire; N/A = Not Applicable. FES-I = Falls Efficacy Scale-International, TUG = Timed Up and Go; TUG-COG = Timed Up and Go-Cognitive. The results are presented as mean ± standard deviation except for the sex variable. [file Table_1.docx]

**Supplementary Table 1. Demographic and Clinical Characteristics**

| Variables | PD group  (n=33) | Healthy controls (n=35) | p-value |  |  |  |  |
| --- | --- | --- | --- | --- | --- | --- | --- |
| Age (years) | 69.3 $\pm$6.7 | 68.5 $\pm$ 6.2 | 0.63 |  |  |  |  |
| Sex (female/male, n) | 14/19 | 21/14 | 0.11 |  |  |  |  |
| Education (years) | 15.30 $\pm$ 2.14 | 17.31$\pm$ 3.53 | 0.006 |  |  |  |  |
| MoCA | 26.61 $\pm$ 3.20 | 26.60$\pm$ 2.31 | 0.99 |  |  |  |  |
| MDS-UPDRS II | 11.91 $\pm$8.23 | N/A | N/A |  |  |  |  |
| MDS-UPDRS III | 43.97 $\pm$ 14.91 | N/A | N/A |  |  |  |  |
| Modified H & Y scale | 2.3 $\pm$ 0.52 | N/A | N/A |  |  |  |  |
| LED (mg) | 302.8$\pm$ 255.7 | N/A | N/A |  |  |  |  |
| SCOPA-AUT | 15.30 $\pm$9.04 | N/A | N/A |  |  |  |  |
| FES-I | 26.39 ± 10 | 18.34 $\pm$ 2.05 | 0.001 |  |  |  |  |
| TUG turning time (sec) | 2.66 $\pm$ 0.55 | 2.27 $\pm$ 0.34 | 0.001 |  |  |  |  |
| TUG total time (sec) | 13.98$\pm$ 4.20 | 11.65$\pm$ 1.83 | 0.004 |  |  |  |  |
| TUG-COG turning time (sec) | 2.69$\pm$ 0.55 | 2.35$\pm$ 0.38 | 0.006 |  |  |  |  |
| TUG-COG total time (sec) | 16.74 $\pm$ 9.75 | 14.44 $\pm$ 5.38 | 0.24 |  |  |  |  |

PD = Parkinson’s disease; MoCA = Montreal Cognitive Assessment; MDS-UPDRS II = Movement Disorder Society Unified Parkinson Disease Rating Scale motor experiences of daily living; MDS-UPDRS III = Movement Disorder Society Unified Parkinson Disease Rating Scale motor examination; H &Y = Hoehn and Yahr; LED = Levodopa Equivalent Dose; SCOPA-AUT = Scales for Outcomes in Parkinson's Disease-Autonomic questionnaire; N/A = Not Applicable. FES-I= Falls Efficacy Scale-International, TUG= Timed Up and Go; TUG-COG= Timed Up and Go-Cognitive. The results are presented as mean ± standard deviation except for the sex variable.
